# Obituary

**Published:** 1920-07

**Authors:** 


### OBITUARY

### DEATH OF D. D. SMITH, D.D.S., M.D.

Dr. D. D. Smith, who has been seriously ill for many years, died at his home in Germantown, Pa., June 3, 1920. Dr. Smith has been such an eminent dental practitioner and teacher for so many years that it is difficult to characterize his tremendous activities. He is so well known because of his enormous contributions to the literature of the profession that it would seem that everybody should know of his works. Dr. Smith was a teacher in the Philadelphia Dental College for several years, but devoted his entire energies for the last twenty years to an earnest study of the subject of treating pyorrhetic teeth and dental prophylaxis. It is well known that Dr. Smith instituted the present system of treating pyorrhea and established the course which he named "Oral Prophylaxis." The course of training which he put into operation, while it has had many apostles, has not been materially modified, although many suggestions have been advocated and adopted by other workers. Those who knew him well became sincerely attached to him personally and became devoted followers of his ideals. The profession will recognize his leadership and credit the strong devotion and his sincere faith in his endeavors to realize that so-called pyorrhea was a curable disease, when others could not believe it possible or desirable to attempt it.

Dr. Smith was born in central New York, but in early boyhood moved to New Hampshire and was essentially a New Englander. He served four years with the Sixteenth Regiment, New Hampshire Volunteers, during the Civil War. In 1866 he entered the Philadelphia Dental College.

Upon graduating he took up the position of professor of mechanical dentistry at the school. He remained a member of the teaching force for fourteen years, and then entered private practice there. While a member of the faculty he attended the Jefferson Medical College and won his degree of Doctor of Medicine in 1878.

Dr. Smith was a member of the American Dental Association, the Massachusetts State Dental Association, the Ontario Dental Society, the New England Society of Pennsylvania, the American Society of Political and Social Science and the Presbyterian Social Union. He attended the Market Square Presbyterian Church.

He is survived by a daughter, Mrs. C. C. Taggart, wife of Dr. C. C. Taggart, of Pittsburg.

